# Antitumor effect and biological pathways of a recombinant adeno-associated virus as a human renal cell carcinoma suppressor

**DOI:** 10.1007/s13277-014-2393-z

**Published:** 2014-08-05

**Authors:** Jie Chen, Xiyun Ruan, Shaomei Wang, Bin Zhang, Bo Liu, Zeqiang Sun, Qingyong Liu

**Affiliations:** 1 Department of Urology, Jinan Central Hospital Affiliated to Shandong University, Jinan, 250013 Shandong China; 2 Department of Neurology, Shandong Provincial Hospital Affiliated to Shandong University, Jinan, 250021 Shandong China; 3 Nephrology and Blood Purification Center, Jinan Central Hospital Affiliated to Shandong University, Jinan, 250013 Shandong China; 4 ICU, Affiliated Hospital of Jining Medical University, Jining, 272129 Shandong China; 5 Department of Urology, Shandong Provincial Qianfoshan Hospital, Shandong University, 16766 Jingshi Road, Jinan, 250014 Shandong China

**Keywords:** Renal cell carcinoma, Recombinant adeno-associated virus, Von Hippel–Lindau, Protein–protein interaction network, Pathway enrichment analysis

## Abstract

The aims of this work are to study the antitumor effect of the adeno-associated virus on the xenografted tumors of chick embryo chorioallantoic membrane and predict potential genes and biological pathways which are associated with renal cell carcinoma. The adeno-associated virus NT4-TAT-6 × His-VHLbeta was constructed and identified. Then, chick embryos with xenografted tumor were divided into three groups and respectively inoculated with rAAV/NT4-TAT-6 × His-VHLbeta (group A), empty virus (group B), and phosphate-buffered saline (group C, the control subject). Antitumor effect in each group was investigated by means of immunofluorescence observation. Genes interacted with von Hippel–Lindau were screened by Search Tool for the Retrieval of Interacting Genes/Proteins database, while pathway analysis were performed based on Kyoto Encyclopedia of Genes and Genomes. The growth of xenografted tumors inoculated with recombinant adeno-associated virus was slower than the control subjects. The tumor volumes of group A showed significant difference compared with group B and group C (*P* < 0.05). Growth of xenografted tumors which administered with the recombinant adeno-associated virus was inhibited. Among the protein–protein interaction network, TCEB2, HIF1A, TCEB1, CUL2, RBX1, and PHF17 were hub genes which might be involved in the development of renal cell carcinoma. The most significant signaling pathway was renal cell carcinoma. In this paper, we constructed and identified the recombinant adeno-associated virus NT4-TAT-6 × His-VHLbeta and studied the antitumor effect of the adeno-associated virus on xenografted tumors of chicken embryo chorioallantoic membrane. In addition, genes in the protein–protein interaction network which are associated with renal cell carcinoma were revealed and the biological pathway of renal cell carcinoma was identified. Our results provide a gene-therapeutic agent for the treatment of human renal cell carcinoma.

**Electronic supplementary material:**

The online version of this article (doi:10.1007/s13277-014-2393-z) contains supplementary material, which is available to authorized users.

## Introduction

Renal cell carcinoma (RCC) is inherently resistant to cytotoxin, hormone, conventional chemotherapy, and radiotherapy [1–3]. Although the response rates of immunotherapeutic and molecular targeting agents are acceptable, their long-term survival benefits remain limited [4]. Approximately 30 %percent of RCC patients develop metastasis [5]. Recently, treatment for patients with metastatic RCC has shifted from conventional cytokines to the angiogenesis inhibitors [6].

Currently, the recombinant adeno-associated virus has been commonly used as a vehicle for delivering therapeutic DNA. This delivery vehicle has the advantages of nonpathogenic nature, broad host range, ability to infect both dividing and nondividing cells, and stabilized expression of transfected genes [7]. Recently, there are many studies on gene expression analysis of RCC. Morris et al. [8] identified tumor methylation of SCUBE3 was associated with a significantly increased risk of cancer death or relapse. While Varela [9] sequenced the protein-coding exome in a series of primary clear cell RCC (ccRCC) and identified the SWI/SNF chromatin remodeling complex gene PBRM1 as a second major gene of ccRCC [10].

Plasmid pBV220-NT4 is a stable and effective vector, which is commonly used as a vehicle for transmitting expression genes. Xi Bao [11] investigated survivin as an anticancer therapeutic target by use of PBV220-NT4 vector. The expression gene of Ant-Shepherdin[79-87] was inserted in PBV220-NT4 vector, and afterward, the gene was sub-cloned into the shuttle plasmid of adeno-associated virus. Zheng et al. [12] inserted NT4-NAP gene into pBV220 to produce a recombinant pBV220-NT4-NAP and transfected it into mammalian retina cells with constant NAP production. The NAP production maintained its biological neuroprotective activities.

Several studies have demonstrated the importance of the von Hippel–Lindau (VHL) gene on inhibiting RCC growth. For example, Lisztwan et al. [13] highlighted that pVHL, the product of the VHL gene, plays an important role in the regulation of cell growth and differentiation of human renal cells. Inactivation of the VHL gene was the frequent genetic event in human RCC and linked to the hereditary VHL disease and sporadic ccRCC [13, 14]. The inactivation of VHL gene predisposed affect individuals to VHL syndrome and was an early genetic event associated with sporadic RCC and CNS hemangioblastomas [15].

Function of pVHL related to its ability to target specific proteins for destruction. The stable complexes of pVHL contained other proteins called Elongin B (TCEB2), Elongin C (TCEB1), Cul2, and Rbx1 [16–18]. These complexes were capable of directing the covalent attachment of polyubiquitin tails to specific proteins, which served as signals for such proteins to be degraded by the proteasome. The VHL tumor suppressor gene had been cloned, and enormous progress were made toward the understanding of the molecular biology and biological function. Over time, germline mutations in VHL patients, as well as somatic mutations in different tumors, were identified, and its ability to act as a tumor suppressor in vivo were confirmed [19]. Several pVHL targets were indicated, including the members of the hypoxia-inducible factor (HIF) alpha family [20]. HIF-1alpha and HIF-2alpha, when bound to a HIF-beta member (such as HIF-1beta), formed a sequence-specific, DNA-binding transcription factor called HIF.

In this article, we constructed the recombinant plasmid pBV220-NT4-TAT-6 × His-VHLbeta and established a human RCC model of chick embryo chorioallantoic membrane. Next, plasmid pBV220-NT4-TAT-6 × His-VHLbeta was sub-cloned into the recombinant adeno-associated virus vector. Afterward, the antitumor effect of the recombinant adeno-associated virus was investigated on xenografted tumors of chicken embryo chorioallantoic membrane. Subsequently, proliferation and apoptotic analyses of transfected cells were performed to further investigate the antitumor effect of the recombinant virus by in vitro assay. Finally, associated functional analysis including protein–protein interaction network and pathway enrichment analysis were performed for VHL genes, and several interactive genes were identified.

## Materials and methods

### Materials

The primers (HisF, HisR; VHL F, VHL R) were synthesized by Shanghai Sangon Co., Ltd. PGEM-T easy carrier and plasmid kits were purchased from Promega Co., Ltd. Construction of the recombinant plasmid pBV220-NT4 were performed by Xi’an Guanghua Biotechnology (China). The restriction endonuclease *EcoR*I, *Eco*72I, *Nae* I and *BamH*I, T4 DNA ligase, QIAquick Gel Extraction kit, and ethidium bromide were all purchased from Huamei Biotechnology (China). Both of the DNA marker and bovine serum albumin were purchased from Gibco Corporation. Taq DNA polymerase and 10 mmol/L dNTP mixed liquor were purchased from MBI Corporation. The 6 × His-Tag antibody was purchased from Abcam.

RLC-310 cells were maintained in DMEM with 100 mL/L fetal calf serum. Fertilized chicken eggs were incubated at 37 °C, relative humidity between 60 and 80 %, air chamber upward.

Both of propidium iodide and 3-(4,5-dimethylthiazol-2-yl)-2,5-diphenyltetrazolium bromide (MTT) were purchased from Sigma Corporation. SR-50 microplate reader was purchased from Pastur Co., Ltd. FACSan440 flow cytometer was purchased from Becton Dickinson, USA.

### Design and synthesis of the primer

DNA sequences were determined according to literature and databases [21], including TAT membrane permeability peptide sequence YGRKKRQRRRD, Label peptide sequence HHHHHH, VHLbeta structure propeptide sequence GTGRRIHSYRGHLWLFRDAG, and fusion posterior sequence YGRKKRQRRRD HHHHHH V GTGRRIHSYRGHLWLFRDAG. The primers were designed according to nucleotide sequence NM 000551 in gene bank.

### Construction and identification of the recombinant plasmid pBV220-NT4-TAT-6 × His-VHLbeta

Generation and ligation of the recombinant plasmids were as follows. The fractions were amplified by PCR, interacting as both primer and template. The first fraction TAT-6 × His was amplified using PCR technique with the forward primer 5′-C GCCGGCGT TATGGCAGGAAGAAGCGGAGACAGCGACGA-3′ and the reverse primer 5′-C GGATCCCACGTGATGATGATGATGATG TCT TCG TCGCTG TC-3′. The second fraction VHLbeta was amplified by PCR using the forward primer 5′-C CAC GTGGGCACGGGCCGCCGCATCCACAGCTACCGAGGTC and the reverse primer 5′-C GGATCCTCACCCTGCATCTCTGAAGAGCCAAAGGT GACCTC GGT AGC.

PCR reaction mixtures contained 10.0 μL 10× PCR buffer, 2.0 μL dNTP mixture (10.0 mmol/L), 3.0 μL of each primer, 81.0 μL double distilled water, and 50.0 μL paroline was added at last.

The PCR program included pre-denaturation at 94 °C for 5 min, 30 amplification cycles each consisting of denaturation at 94 °C for 1 min after adding 1.0 μL Taq DNA polymerase, annealing at 31 °C for 1 min (the second fraction annealing at 35 °C for 1 min), and extension at 72 °C for 1 min, followed by further extension at 72 °C for 5 min.

The PCR product were purified and combined to the vector with T4 DNA ligases. The TAT-6 × His PCR product was digested with *Nae* I and *BamH*I restriction enzymes, while the VHLbeta PCR product was digested with *Eco72*I and *BamH*I restriction enzymes. Both of the DNA fragments after digestion were cloned into plasmid pGEM-T easy.

Therecombinant plasmid was amplified in *Escherichia coli* DH5alpha competent cells using CaCl2 method. NT4-TAT-6 × His-VHLbeta transformants were selected on Luria-Bertani agar containing ampicillin.

Plasmid preparation was performed by using the alkaline lysis method [21]. The products were digested with *EcoR*I and analyzed by electrophoresis in 10 g/L agarose with staining by ethidium bromide. Authenticity of all DNA fragments was confirmed by DNA sequencing.

The recombinant plasmid was identified by the restriction digestion analysis [21]. The plasmids pGEM-T-TAT-6 × His and pGEM-T-VHLbeta were respectively digested with *BamH*I and *Eco72*I restriction enzymes. Afterward, pGEM-T-TAT-6 × His with a cohesive-end and the target DNA fragment VHLbeta were combined together with T4 DNA ligases overnight. Then, the recombinant plasmid pGEM-T-TAT-6 × His-VHLbeta was digested with *EcoR*I restriction enzymes, and DNA fragments were collected for DNA sequencing.

**Fig. 1:**
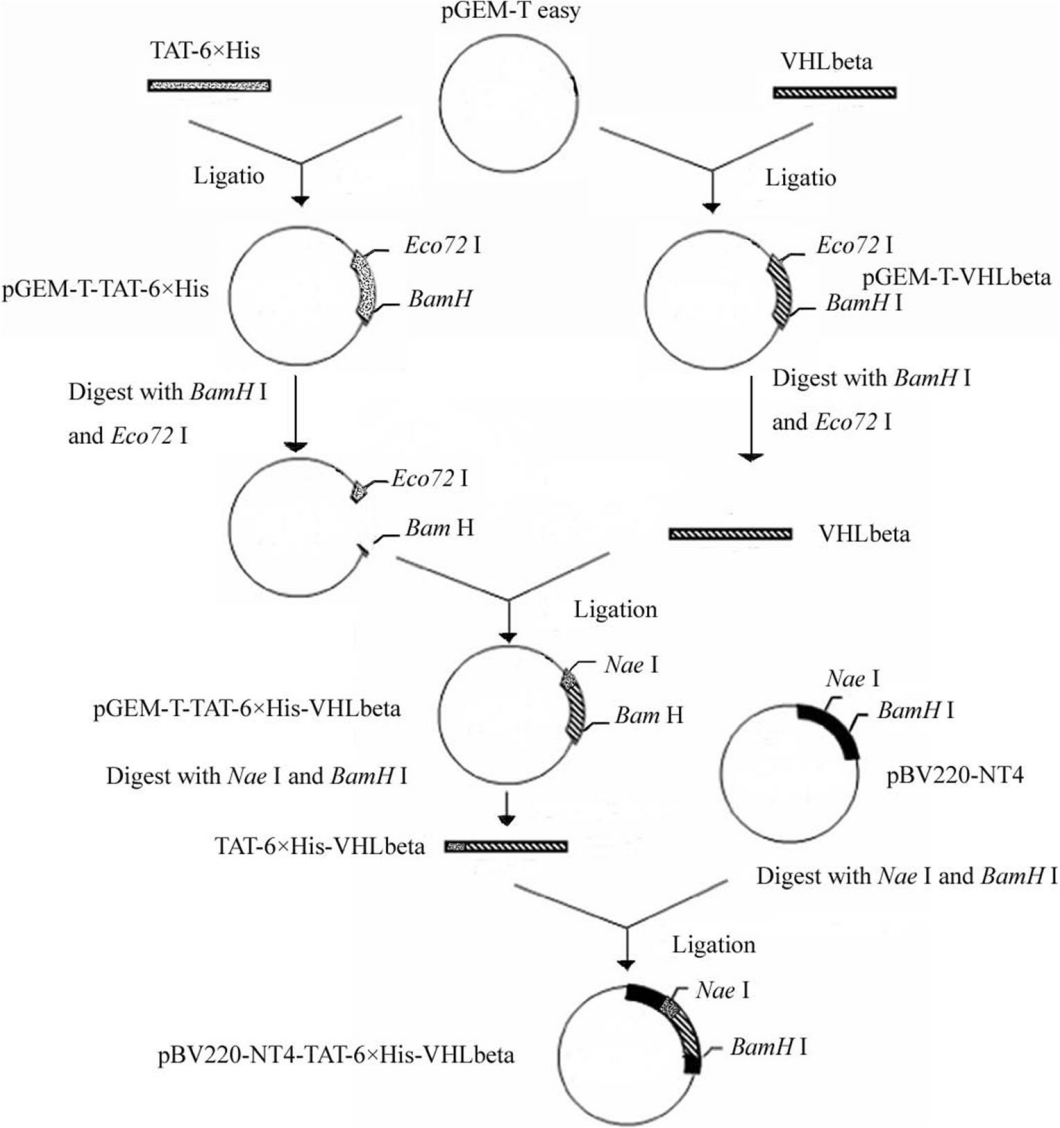
Diagram of the constructed recombinant plasmid pBV220-NT4-TAT-6 × His-VHLbeta

The recombinant plasmid pBV220-NT4-TAT-6 × His-VHLbeta was constructed as follows: The vector pBV220-NT4 and plasmids pGEM-T-TAT-6 × His-VHLbeta complementary DNA (cDNA) were respectively digested with *Nae* I and *BamH*I. Afterward, the plasmids pBV220-NT4 with a cohesive-end and the target DNA fragment VHLbeta were combined together with T4 DNA ligases overnight. Then, the recombinant plasmid pBV220-NT4-TAT-6 × His-VHLbeta was digested with *BamH*I and *EcoR*I, and DNA fragments was collected for identification of the recombinant plasmids. Construction of plasmid pBV220-NT4-TAT-6 × His-VHLbeta is shown schematically in Fig. 1.

### Establishment of renal cell carcinoma model on chicken embryos chorioallantoic membrane

Forty chicken embryos incubated for night days were randomly divided into five groups, with eight embryos in each group. Before xenografting into chicken embryo chorioallantoic membrane (CAM), RLC-310 cells were washed twice with sterile phosphate-buffered saline (PBS). Then, RLC-310 cell lines in 30-μL PBS at different cell concentration (1.0, 2.0, 4.0, 8.0 × 10^6^ PFU/30 μL) were deposited on the avascular zone of the intact chicken embryo CAM in four groups, respectively. The last group was defined as the control subject which inoculated 30-μL PBS. Chicken embryos were sealed with sterile scotch tape after inoculation.

Tumor growth was monitored 7 days after inoculation to determine the optimum cell concentration for high tumor rate. Tumor was regarded as positive when the diameter was 2 mm or greater. Angiogenesis of the tumors were observed 5 days after inoculation. After formaldehyde fixation, paraffin embedding, and section cutting, the xenografted tumor tissue was observed under a microscope, with a method of hematoxylin–eosin staining.

### Effect of the recombinant adeno-associated virus on xenografted tumor

The recombinant adeno-associated virus was added to the chick embryo model. Subsequently, bioactivities of the recombinant adeno-associated virus were identified by tumor suppression assay.

Three days after the inoculation of RLC-310, chicken embryos with xenografted tumor were divided into three groups. Group A was administered with 100-μL recombinant adeno-associated virus at the concentration of 1.0 × 10^9^ PFU/100 μL on the xenografted tumors, group B with 100-μL empty virus at the same concentration, and group C with 100-μL PBS.

Five days after the inoculation, the tumor volume was estimated using the following formula:

$$ V\left({\mathrm{mm}}^3\right)=0.40\times {a}^2\times b $$

where *V* (mm^3^) is the tumor volume, *a* (m) is the long diameter of the tumor, and *b* (m) is the short diameter of the tumor.

Expression of the recombinant adeno-associated virus was confirmed using immunofluorescence with the 6 × His-Tag antibody, labeled by fluorescein isothiocyanate. Cell lines were stained by immunofluorescence, and detailed information of immunofluorescence was described by Gardner and McQuillin [22] and Fenwick et al. [23]. Changes of the xenografted tumor were observed through immunofluorescence observation. Xenografted tumor was regarded as positive if cells in experimental groups responded positively in immunofluorescence tests (++), while those of control subject did negatively (±or −). After formaldehyde fixation, paraffin embedding, and section cutting, the xenografted tumor tissue was observed under a microscope, with a method of hematoxylin–eosin staining.

### Proliferation and apoptotic analysis of RLC-310 cells

The effect of recombinant adeno-associated virus on RLC-310 cell proliferation was measured by a colorimetric MTT assay. The proliferation of RLC-310 cells, which induced the intensity of color development was examined by colorimetric method using microplate reader according to the manufacturer’s instructions.

The effect of recombinant adeno-associated virus on RLC-310 cell apoptosis was analyzed with flow cytometry using flow cytometer. Apoptotic was detected using staining of the cells with propidium iodide, as described by Vermes et al. [24]. Detailed information on method of the proliferation and apoptotic assay is shown in the supplementary material.

### Protein–protein interaction network construction

Biological modules can be approximately reflected by gene sets. To identify genes which play a vital role with VHL, the protein–protein interaction (PPI) network was constructed. The PPI data were downloaded from Search Tool for the Retrieval of Interacting Genes/Proteins (STRING) (http://string.embl.de/). The VHL gene were mapped into the interaction network, and genes interacted with VHL were screened. The networks were constructed using Cytoscape software.

### Pathway enrichment analysis

To further investigate the biological functions of these genes, the Kyoto Encyclopedia of Genes and Genomes (KEGG) pathway enrichment analysis for modules were performed by using the online tool Database for Annotation, Visualization, and Integrated Discovery (DAVID) [25]. Biological meaning could be systematically extracted from a large number of genes or proteins using DAVID bioinformatics resources containing an integrated biological knowledgebase and analytic tools.

### Statistical analysis

The computer-based analysis program SPSS 13.0 was used for statistical analyses. The rate and volume of the tumors between different groups was respectively assessed by analysis of *χ* ^2^ and ‾x ± s. Variance with *P* < 0.05 was regarded as statistically significant. Comparison between different groups was assessed by least significant difference. The EASE score was used to detect the significant categories in the functional enrichment and pathway enrichment analysis [26]. The threshold of EASE score <0.01 and the minimum number of genes for the corresponding term >2 were considered significant for a category.

## Results

### Construction and identification of the recombinant plasmid pBV220-NT4-TAT-6 × His-VHLbeta

**Fig. 2:**
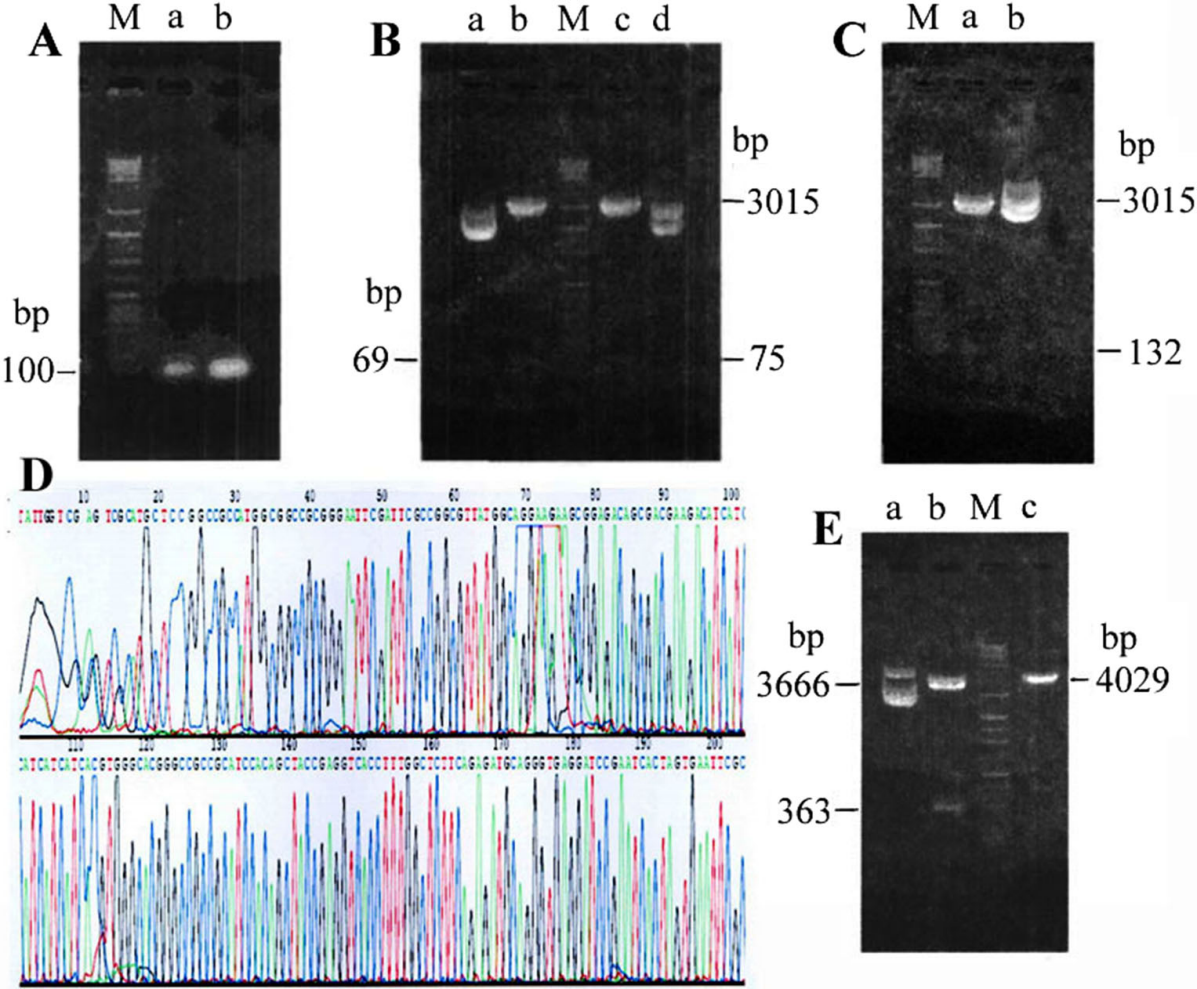
Validation of the recombinant plasmid pBV220-NT4-TAT-6 × His-VHLbeta. **a** Validation of PCR products of TAT-6 × His and VHLbeta cDNA; DNA marker (*M*); PCR product of TAT-6 × His (*a*); PCR product of VHLbeta cDNA (*b*). **b** Validation of recombinant plasmid pGEM-T-TAT-6 × His and pGEM-T-VHLbeta by restriction enzyme digestion; DNA marker (*M*); pGEM-T-TAT-6 × His (*a*); pGEM-T-TAT-6 × His/*EcoR*I (*b*); pGEM-T-VHLbeta/*EcoR*I (*c*); pGEM-T-VHLbeta (*d*). **c** Validation of recombinant plasmid pGEM-T-TAT-6 × His-VHLbeta by restriction enzyme digestion; DNA marker (*M*); pGEM-T-TAT-6 × His-VHLbeta/*Nae* I + *BamH*I (3,015 + 132 bp) (*a*); pGEM-T-TAT-6 × His-VHLbeta (without enzyme digestion) (*b*). **d** Partial sequencing of recombinant plasmid pGEM-T-TAT-6 × His-VHLbeta. **e** Validation of recombinant plasmid pBV220-NT4-TAT-6 × His-VHLbeta by restriction enzyme digestion; DNA marker (*M*); pBV220-NT4-TAT-6 × His-VHLbeta (*a*); pBV220-NT4-TAT-6 × His-VHLbeta/*EcoR*I + *BamH*I (3,666 + 363 bp) (*b*); pBV220-NT4-TAT-6 × His-VHLbeta/*EcoR*I (4,029 bp) (*c*)

The expected length of NT4-TAT-6, VHLbeta cDNA, and the *EcoR*I digested pGEM-T fragment were 69, 75, and 3,015 bp, respectively. The agarose gel electrophoresis analysis showed that after *BamH*I*-Eco72*I double digestion, both of the NT4-TAT-6-digested fragment and the VHLbeta-digested fragment left were no more than 100 bp (Fig. 2a). Moreover, the agarose gel electrophoresis analysis of the digested fragments from both of pGEM-T-TAT-6 × His and pGEM-T-VHLbeta cDNA also showed the consistent length as expected (Fig. 2b).

The inserted fragments with target gene (132 bp) from recombinant plasmid pGEM-T were verified by agarose gel. The VHLbet*a* cDNA was successfully inserted into pGEM-T-TAT-6 × His plasmid identified by *BamH*I digestion (Fig. 2c), while the partial sequencing analysis showed the TAT-6 × His-VHLbeta cDNA were consistent with the sequences as reported by Datta et al. [27] (Fig. 2d).

The NT4-TAT-6 × His-VHLbeta cDNA (363 bp) from recombinant plasmid pBV220-NT4-TAT-6 × His-VHLbeta was also verified by agarose gel. The VHLbeta cDNA was successfully inserted into pBV220-NT4-TAT-6 × His-VHLbeta plasmid (Fig. 2e), identified by *EcoR*I and *BamH*I double digestion (3,666 bp) and *EcoR*I digestion (4,029 bp).

### Establishment of renal cell carcinoma model on chicken embryos chorioallantoic membrane

Inoculation of RLC-310 cell lines on the chick embryos resulted in the development of large tumors. Table 1 showed a strong relationship between tumor rate and RLC-310 cell concentration on the seventh day after inoculation. The more cell concentration it inoculated, the higher tumor rate goes. Comparison was conducted with the analysis of variance, which showed significant difference between each group (*P* < 0.05).

**Table 1:** Effects of the inoculated cell concentration on tumor rate

Group	Cell concentration (×10^6^ PFU/30 μL)	Number of the chick embryos	Positive [*n* (%)]
A	0	8	0 (0)
B	1	8	1 (12.5)
C	2	8	3 (37.5)
D	4	8	7 (87.5)
E	8	8	8 (100)

Group B showed significant difference compared with group D and E (*P* < 0.05), while there were no significant difference between group E and group D (*P* > 0.05), although the tumor rate of group E was higher than group D.

The tumor, with an average diameter of 6 mm, exhibited an ivory-white appearance on the surface and integrated into the mass of the CAM. CAM of the control group showed no significant change after inoculation.

**Fig. 3:**
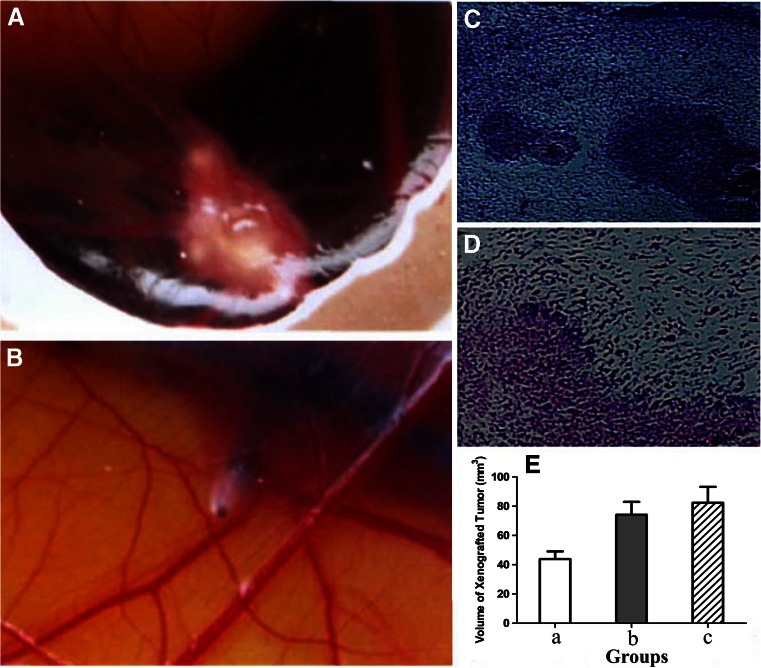
Evaluation of xenografted tumors on CAM. **a** A photograph of the xenografted tumor on the fifth day after inoculation. **b** A photograph of blood vessels of the control group on the fifth day after inoculation. **c** A microscopic evaluation of the xenografted tumor (HE, ×100). **d** A microscopic evaluation of the xenografted tumor (HE, ×200). **e** Volume of the xenografted tumor in each group (mm^3^); chick embryos administered with rAAV/NT4-TAT-6 × His-VHLbeta (*a*); empty virus (*b*); control (*c*)

On the fifth day after inoculation, the blood vessel distributed in and around the xenografted tumor (Fig. 3a), while blood vessel of the control group, which exhibited a very smooth surface in a form of arborization, showed no significant change after inoculation (Fig. 3b).

On the seventh day after inoculation, the chicken embryos showed cyst formation and focal necrosis. The cancer cells showed a diversity arrangement and various appearances, which adopted a bulky and polygonal shape (Fig. 3c, d).

### Effect of the recombinant adeno-associated virus on xenografted tumor

**Fig. 4:**
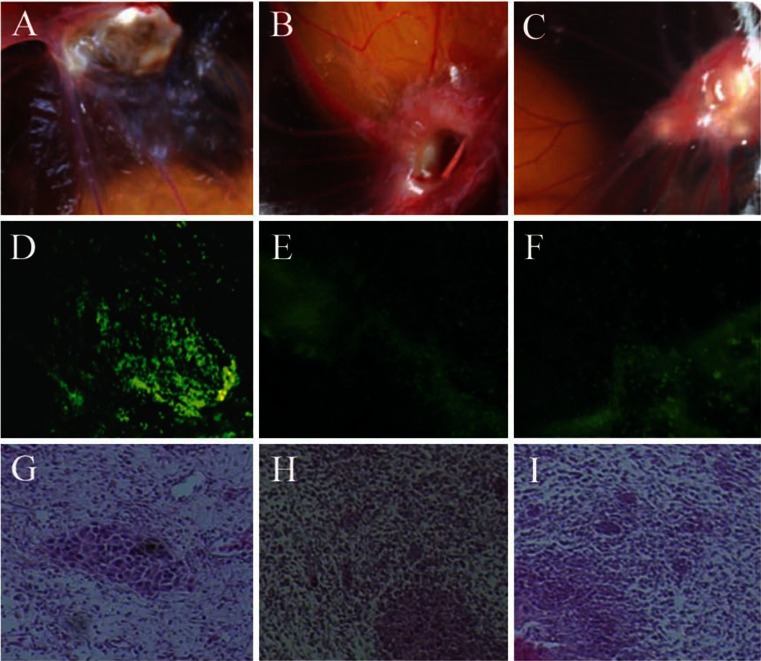
Evaluation of xenografted tumors administered with different reagents. **a** A photograph of the xenografted tumor administered with rAAV/NT4-TAT-6 × His-VHLbeta. **b** A photograph of the xenografted tumor administered with empty virus. **c** A photograph of the xenografted tumor of the control group. **d** An immunofluorescence evaluation of the xenografted tumor administered with rAAV/NT4-TAT-6 × His-VHLbeta (×100). **e** An immunofluorescence evaluation of the xenografted tumor administered with empty virus (×100). **f** An immunofluorescence evaluation of the xenografted tumor of the control group (×100). **g** A microscopic evaluation of the xenografted tumor administered with rAAV/NT4-TAT-6 × His-VHLbeta (HE, ×200). **h** A microscopic evaluation of the xenografted tumor administered with empty virus (HE, ×200). **i** A microscopic evaluation of the xenografted tumor of the control group (HE, ×200)

Photographs of xenografted tumors are shown in Fig. 4a–c. Blood vessel in each group were radiating outward in the tumor. Tumors in group A showed unsharp images, while those in group B and group C were concentrated and clearly visible.

Comparison was conducted with the analysis of variance, which showed significant difference between each group (*F* = 4.72, *P* < 0.05). The tumor volumes of group A was significantly reduced when compared with group B and group C (*P* < 0.05). Although the tumor volume of group C was greater than that of group B, there were no significant differences between them (*P* > 0.05). The tumor volumes are shown in Fig. 3e.

Immunofluorescence images of xenografted tumors are shown in Fig. 4d–f. Bright fluorescent appeared in group A (++), while no fluorescence was found in group B and group C (−).

Microscope images of xenografted tumors are shown in Fig. 4g–i. In the epithelial tissue of chick embryos in group A, tumor mass and tissue around seemed to dissect into separate areas with the spontaneous percept of distinct borders between them.

### Proliferation and apoptotic analysis of RLC-310 cells

The effect of recombinant virus on proliferation of RLC-310 cells is shown in Fig. S1. The proliferation of RLC-310 cells that administered with recombinant virus was significantly restrained compared to the two control groups.

The effect of recombinant virus on apoptosis of RLC-310 cells is shown in Fig. S2. The apoptotic rate of RLC-310 cells that administered with recombinant virus was significantly raised compared to the two control groups (detailed information on result of the proliferation and apoptotic assay is shown in the supplementary material).

### Protein–protein interaction network construction

**Fig. 5:**
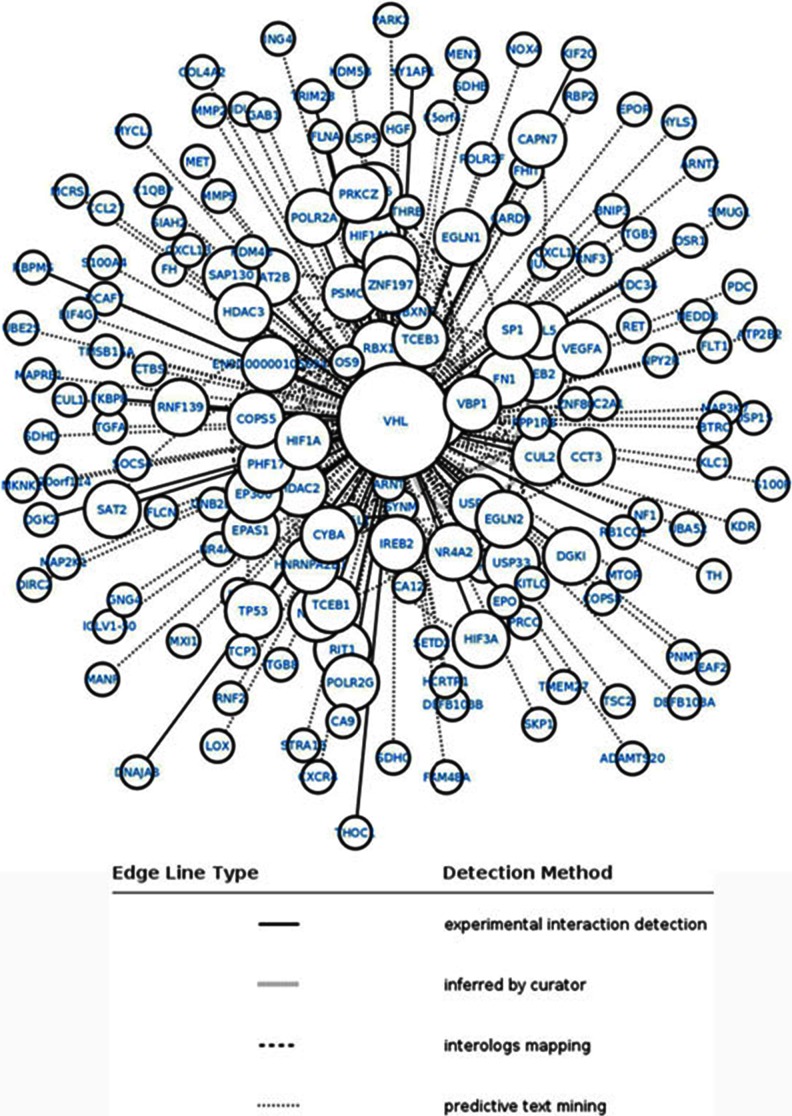
The PPI network consisting of 170 genes related to VHL (VHL is the central node). The pertinent interaction is between the central node and the edges. Types of edge lines which stand for different detection methods are shown in the figure

To obtain a PPI network, the relationship of proteins encoded by 170 genes associated to VHL were matched according to the PPI database. By integrating interaction relationships, a PPI network was constructed (Fig. 5). Genes at the top of degree distribution in the significantly perturbed networks were defined as hub genes. Hub genes with degree equal or greater than three in the PPI network are shown in Table 2. Among the nodes in perturbed networks, we found night hub genes with the highest degree of structural disorder: EPAS1, TCEB2, HIF1A, TCEB1, HDAC2, CUL2, EGLN2, RBX1, and PHF17.

**Table 2:** The genes that degree equal or greater than three in PPI network

Degree	Genes
VHL	230
EPAS1	4
TCEB2	4
EGLN2	3
HIF1A	4
TCEB1	4
HDAC2	4

### Pathway enrichment analysis

**Fig. 6:**
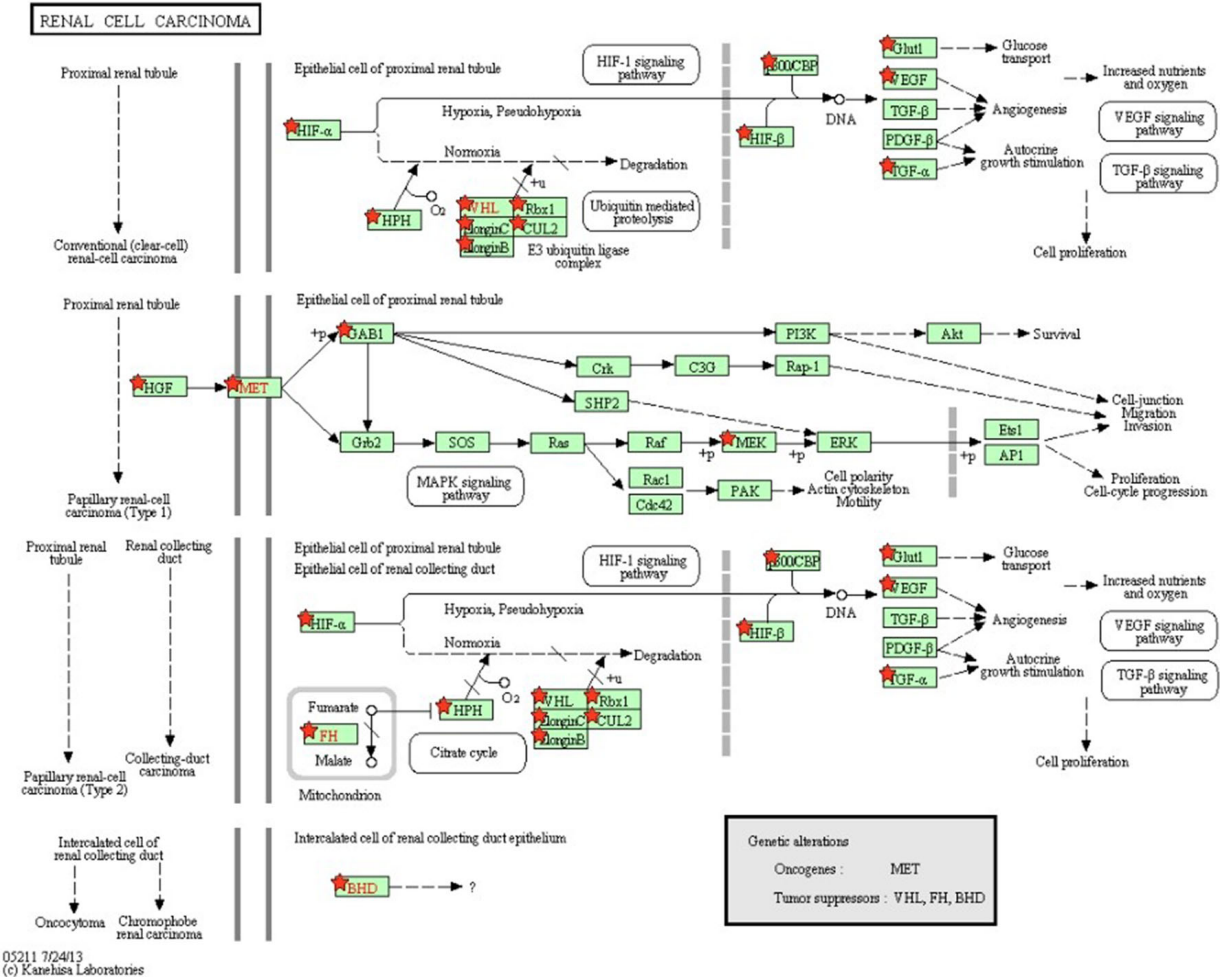
The KEGG pathway of renal cell carcinoma consisting of 170 genes related to VHL. Both of the first and the third routes of renal cell carcinoma pathway are activated. Notation of the pathway map is shown in the figure

Pathway enrichment analyses were carried out for these genes. Results showed that 170 genes in PPI network were significantly enriched in seven pathways (Table 3). The most significant signaling pathway was renal cell carcinoma (*P* = 0.81511671), which is shown in Fig. 6. Genes such as TCEB2, HIF1A, TCEB1, CUL2, and RBX1 were involved in both of the first and the third routes of the renal cell carcinoma pathway. Both of the molecular interaction and the missing interaction were expressed, suggesting that these two routes were activated in the RCC process.

**Table 3:** The pathways analysis based on KEGG

Term	*P* value	Genes
hsa05211:Renal cell carcinoma	2.61E − 21	MAP2K1, EPAS1, VHL, ARNT2, MET, EGLN3, EGLN2, EGLN1, HGF, FLCN, ARNT, RBX1, CUL2, EP300, HIF1A, VEGFA, GAB1, SLC2A1, TCEB2, TGFA, TCEB1, FH
hsa05200:Pathways in cancer	6.05E − 13	MMP9, ARNT2, EGLN3, EGLN2, KITLG, EGLN1, MMP2, ARNT, RBX1, CUL2, SLC2A1, TGFA, FH, FN1, RET, COL4A2, EPAS1, MAP2K1, VHL, MET, TP53, HGF, HIF1A, HDAC2, EP300, VEGFA, TCEB2, MTOR, TCEB1
hsa04120:Ubiquitin mediated proteolysis	6.21E − 07	VHL, BTRC, CDC34, PARK2, SKP1, RBX1, CUL2, CUL5, TCEB2, SIAH1, TCEB1, ITCH, UBE2S, CUL1
hsa04510:Focal adhesion	0.002691788	COL4A2, FLT1, MAP2K1, ITGB8, MET, VEGFA, ITGB5, HGF, FLNA, KDR, FN1
hsa05016:Huntington’s disease	0.004264142	SDHB, POLR2G, POLR2F, EP300, HDAC2, SP1, SDHC, SDHD, TP53, POLR2A
hsa04060:Cytokine–cytokine receptor interaction	0.00610182	FLT1, CXCR4, CXCL13, MET, VEGFA, KITLG, EPOR, HGF, CXCL12, CCL27, KDR, EPO
hsa05219:Bladder cancer	0.006148358	MAP2K1, MMP9, VEGFA, TP53, MMP2

## Discussion

In this study, we established a stable and highly effective method of delivering gene construct containing VHL cDNA into RLC-310 cells as a way of attaining continuous VHL production. Recombinant adeno-associated virus was assembled to achieve this goal. The antitumor effect of the recombinant adeno-associated virus on RCC was demonstrated. Xenografted tumors as well as transfected cells which administered with the recombinant adeno-associated virus resulted in growth inhibition, indicating that the adeno-associated virus might be used as a gene-therapeutic agent for the treatment of RCC.

In addition, we found a series of hub genes involved in PPI network which interacted with VHL and predicted the statistically significant KEGG pathway of renal cell carcinoma. Moreover, the significance of those indicated hub genes in the present study was further confirmed by the significant signaling pathway of RCC. As shown in Fig. 6, TCEB2, HIF1A, TCEB1, CUL2, and RBX1 were involved in the renal cell carcinoma pathway, while TCEB2, HIF1A, TCEB1, and CUL2 had the highest degree in the PPI network. This suggested that those indicated hub genes might be highly related to the development of RCC. All these results indicated that TCEB2, HIF1A, TCEB1, and CUL2 might be related with RCC. In addition, the influence of HIF1A [28–31], TCEB1 [32], CUL2 [33], and PHF17 [34] on development of RCC had been reported, which verified the inference above.

The pVHL complex has the ability to ubiquitinate proteins which can then mark these proteins for subsequent degradation by the proteasomal machinery of the cell [13, 35]. This ubiquitin ligase activity was one of the critical mechanisms by which the cell regulated the protein level of key regulatory molecules in the cell, such as HIF1-alpha [36]. Hypoxia inducible factor 1 (HIF-1) was a key regulator of the genes involved in the cellular response to hypoxia [37]. It was subsequently demonstrated that this occurred through the oxygen-dependent polyubiquitination and subsequent degradation of HIF-alpha that was critically mediated and regulated by the VHL protein [29–31, 38]. The VHL and HIF1-alpha polymorphisms might jointly influence RCC progression and survival [28].

Sequences outside the TCEB1 binding box might function as a nuclear export domain, potentially providing a novel role for this region of VHL frequently mutated in RCC. Inactivating mutations of the VHL tumor suppressor gene caused the VHL cancer syndrome and sporadic ccRCC [32]. In addition, defective VHL-mediated proteolysis as a common feature of ccRCC was caused not only by VHL inactivation but also by new hotspot TCEB1 mutations, which abolished TCEB1–VHL binding, leading to HIF accumulation [39].

As binding of the VHL gene product to the CUL2 protein is important for pVHL function, germline CUL2 mutations were searched. Although no pathogenic mutations were detected, CUL2 polymorphisms were identified [33]. A model for the regulation of hypoxia-inducible mRNAs by pVHL was respectively presented based on the apparent similarity of TCEB1 and Cul2 to Skp1 and Cdc53. These latter proteins formed complexes that target specific proteins for ubiquitin-dependent proteolysis [40], suggesting that CUL2 takes part in the development of RCC.

PHF17 could suppress RCC in part by increasing apoptosis. Most renal cancers had defects in the VHL tumor suppressor pVHL. PHF17 was a short-lived, kidney-enriched transcription factor that was stabilized by direct interaction with pVHL. Loss of PHF17 stabilization with pVHL correlated with renal cancer risk, suggesting the relationship between PHF17 and renal cancer. In addition, PHF17 was a candidate transcriptional co-activator which might play a key role in the pathogenesis of renal cancer and VHL disease [34]. In conclusion, PHF17 was identified as a protein partner of the VHL tumor suppressor pVHL.

The CAM assay was well established and widely used as a model for tumor angiogenesis and invasiveness. In 1976, Knighton et al. [41] first implanted carcinosarcoma on the chrioallantoic membrane. Currently, CAM has been used in research involving angiogenesis, microcirculation, oncobiology, etc [42]. In this article, we established a human RCC model on CAM, and the antitumor effect of the recombinant adeno-associated virus had been demonstrated experimentally on CAM xenografted tumor. Major findings developed from the CAM agents were accomplished.

The minigene anticancer therapy with the application of the recombinant adeno-associated virus will be used in the clinic. However, further validation is required to elucidate the expression of hub gene in protein or RNA level that involved in development of renal cell carcinoma. Furthermore, the security and specificity of the recombinant adeno-associated virus is to be determined. Therefore, such gene therapy would still be far from clinical use.

## Conclusion

In this paper, we constructed and identified the recombinant adeno-associated virus NT4-TAT-6 × His-VHLbeta and revealed the relevant biological function related to RCC. Based on the network analyses, we identified a series of hub genes which might be involved in the development of RCC. Transfection of recombinant virus in RLC-310 cells strongly restrained cell proliferation and markedly induced apoptosis. Genes of TCEB2, HIF1A, TCEB1, and CUL2 which participated in renal cell carcinoma pathway might be the potential target genes for RCC treatment.

The results of this study demonstrated the feasibility of the recombinant adeno-associated virus with VHL gene, also suggesting that such a construct may provide the basis for future clinical applications in treatment of RCC.

## Electronic supplementary material

Below is the link to the electronic supplementary material.

ESM 1 (DOCX 221 kb)
