# Supplementary material for: Antitumor effect and biological pathways of a recombinant adeno-associated virus as a human renal cell carcinoma suppressor
Source: Tumour Biol. 2014 Aug 5;35(11):10993–1003. doi: 10.1007/s13277-014-2393-z (PMC4244535; doi:10.1007/s13277-014-2393-z)
Supplement: Supplementary file 1 — (DOCX 221 kb) [file 13277_2014_2393_MOESM1_ESM.docx]

**Supplementary Materials: Proliferation and apoptotic assay of RLC-310 cells**

**1. Methods**

*1.1 Proliferation analysis of RLC-310 cells*

For the proliferation assay, RLC-310 cells were freshly isolated and plated in 96-well flat bottom tissue culture plate at a concentration of 4×10^4^ cells/well containing 100 µL tissue culture medium. After an overnight incubation at 37℃, cells were administered with 100 µL/well recombinant adeno-associated virus and 1 µL/well polybrene. The positive control cells were administered with empty virus, while the control cells were treated with phosphate-buffered saline (PBS), at the same concentration. At 0, 24, 48 and 72 h of culture, 20 µL of 5 mg/mL 3,(4, 5-dimethyl-2-thiazolyl)- 2, 5-diphenylte -2H-tetrazolium bromide (MTT) solution was respectively added per well. After 4 h of incubation at 37℃, colored crystals were dissolved with a 150 µL dimethylsulfoxide of dissolving solution. Plates were kept on orbital shaker for 10 min and optical density was read on a microplate reader at 490 nm for wavelength, and 630 nm for reference wavelength.

*1.2 Apoptotic analysis of RLC-310 cells*

Before the apoptotic assay, RLC-31cells were respectively transfected with recombinant adeno-associated virus, empty virus as positive control and PBS as the control. Subsequently, cells were rinsed in phosphate buffer for twice, then cells were resuspended at 1×10^6^/mL in combine buffer. Next, 100 µl cells were stained with 5 µL propidium iodide and incubated [in](javascript:void(0);) [dark](javascript:void(0);) for 15 min at room temperature . Afterwards, the cells were mixed with 400 µl dye buffer, and analyzed with flow cytometry using flow cytometer.

*1.3 Statistical Analysis*

The data were quantified with the computer-based analysis program SPSS 13.0. Data in all groups was assessed by analysis of ‾x±s. Comparation between different groups was assessed by [variance](javascript:void(0);) [analysis](javascript:void(0);). Comparation within groups was assessed by t text. Variance with *P*<0.05 was regarded as statistically significant.

**2. Results**

*2.1 Proliferation analysis of RLC-310 cells*

The effect of recombinant adeno-associated virus on proliferation of RLC-310 cells measured by MTT is shown in Figure S1. In recombinant adeno-associated virus administered RLC-310 cells, the optical density were significantly lower than the control groups (*P* ＜ 0.05). There is no significant difference (*P* > 0.05) in the optical density between the empty virus group and the control group. The proliferation of RLC-310 cells that administered with recombinant adeno-associated virus were significantly restrained compared with the two control groups.

*2.2 Apoptotic analysis of RLC-310 cells*

The effect of recombinant adeno-associated virus on apoptotic of RLC-310 cells analyzed with flow cytometry is shown in Figure S2. In the control group, only 4.58 % cells were apoptotic; in the empty virus group, 8.09% cells were apoptotic, while in the virus transfected group, 36.20% were apoptotic. There is no significant difference (*P* > 0.05) in the apoptotic rate between the empty virus group and the control group. While in the virus administered RLC-310 cells, the apoptotic rate were significantly higher than the control groups (*P* ＜ 0.01). The apoptotic rate of RLC-310 cells that administered with recombinant adeno-associated virus were significantly raised compared to the empty virus group and the control group.

**3. Conclusion**

For RLC-310 cells that transfected with recombinant virus, cell proliferation was significantly restrained, while the apoptotic rate of them was significantly raised, compared to the empty virus group and the control group. In conclusion, the present study shows that transfection of recombinant adeno-associated virus in RLC-310 cells strongly restrained cell proliferation and markedly induced apoptotic.

**Supplementary Figures**


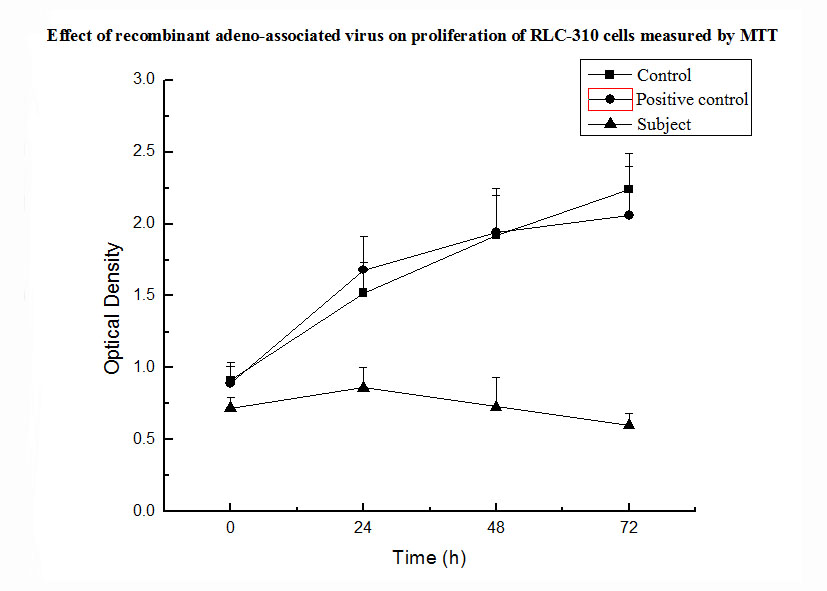


Figure S1: Effect of recombinant adeno-associated virus on proliferation of RLC-310 cells measured by MTT


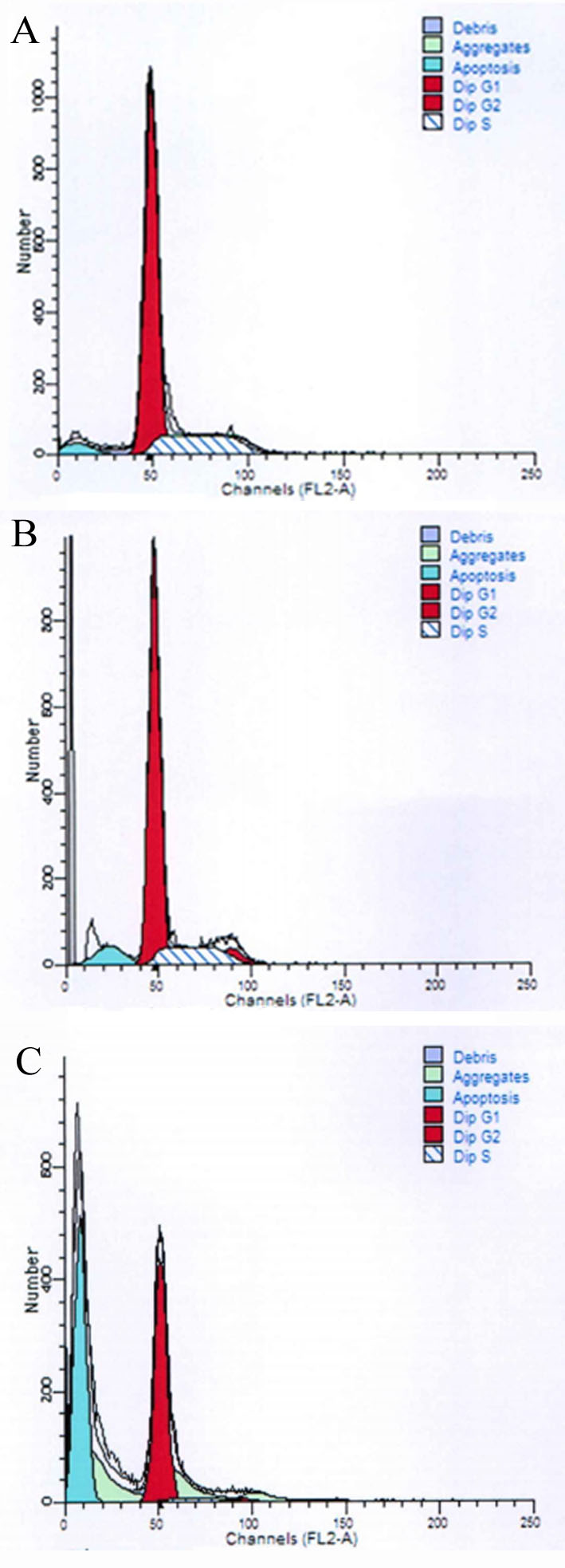


Figure S2: Apoptotic analysis of RLC-310 cells (A: Control; B: Empty virus; C: Recombinant adeno-associated virus transfected)
